# Profiles of Wellbeing in Soft and Hard Mountain Hikers

**DOI:** 10.3390/ijerph19127429

**Published:** 2022-06-17

**Authors:** Piotr Próchniak

**Affiliations:** Institute of Psychology, University of Szczecin, 71-017 Szczecin, Poland; piotr.prochniak@usz.edu.pl

**Keywords:** outdoor recreation, hiking, adventure, wellbeing

## Abstract

The aim of the study was to analyze the wellbeing profiles in a group of Polish mountain hikers. The study involved 242 young people (M = 23.50; SD = 4.40) who completed various wellbeing scales: The *Oxford Happiness Questionnaire (OHQ)*, *Meaning of Life Questionnaire (MLQ)*, *Positive and Negative Affect Scale (PANAS*), *Time Satisfaction Scale (TSS)*, *Hope Scale*, *General Self-Efficiency Scale*, *Ego Resiliency Scale*, *Revised Life Orientation Test (LOT-R)*, and *Adventure-Seeking Behavior Scale*. Cluster analyses revealed two types of mountain hikers: hard adventure hikers and soft adventure hikers, with different profiles of subjective wellbeing. Hard adventure hikers most often revealed high levels of life satisfaction, control of life, meaning of life, and positive emotions, along with low levels of negative emotions. Moreover, these hikers revealed high levels of satisfaction in various time perspectives (past, present, and future) and a high level of psychological capital. On the other hand, soft adventure hikers most often revealed an average level of satisfaction with life, control of life, and positive emotions, average satisfaction in the past and present time perspective, and average levels of psychological capital. Soft adventure hikers also revealed higher levels of negative emotions and satisfaction in the future time perspective. The present research indicated that mountain hikers are not a homogenous group. The profiles of wellbeing in the hikers varied depending on the type of stimulating behavior in a natural environment.

## 1. Introduction

Hiking is a widely used term with a broad definition. Hiking involves walking in a natural environment on an established trail. There are day hikes and overnight hikes. A hike may last 1 h or more; moreover, it may be a simple outback trail, a loop hike, or a point-to-point hike. The terrain can vary from flat to very steep. Hiking tours include short hikes and daily excursions [1,2,3].

One of the most popular types of hiking is mountain hiking. Mountain hiking is the activity of going for long walks in mountainous areas with altitude differences. Therefore, mountain hikers must be in good physical condition. Sometimes, specific equipment is needed (e.g., helmet, crampons, axes, clip hooks, ropes, and poles) in order to mountain hike, which reduces the risk of accidents associated with mountain hiking [4,5,6].

Mountain hiking may include forms associated with a low possibility of accidents including those in which the risk, understood as the possibility of losing health or even life, is significant. A walk in a valley between low mountains is an example of high-safety hiking. However, hiking in the Alps, Dolomites, Himalayas, or other high mountains can be associated with a high risk. Hazards in such cases are connected with injuries from falls on the trail. In addition, strong winds, heavy rain, fog, dust, or large amounts of mud can place a significant mental and physical strain on hikers. Additionally, hiking in winter may be associated with intense cold, ice on the trail, high winds, or avalanches. Snowfall may limit visibility, erases track, and cover trail markings [7,8,9].

According to terminology of Hill and Millington et al. [10,11], I distinguish between hard and soft adventure hiking. Hard adventure hiking requires high skills, preparation, experience, and sometimes specialized equipment (such as mountain ice axes or harnesses) because the environmental dangers are quite high. Soft adventure hiking does not require competences and specialized equipment. This distinction is congruent with the classifications of hiking proposed by the Italian and Swiss Tourist Clubs. These clubs distinguish different types of hiking in mountains: mountain hiking, challenging mountain hiking, and alpine hiking. Different types of hiking are connected with different levels of difficulty: from marked trails with an easy terrain that is physically less demanding to usually unmarked trails with terrain that is very exposed, challenging, and dangerous [12,13,14].

The dangers of mountain hiking are a natural aspect of exploring the wilderness; however, we can also observe several benefits which can overshadow the hazards. The main health benefits of mountain hiking include losing excess weight, regulating blood pressure, body fat, body mass index, and total cholesterol, improving sugar levels, and building strength in muscles. Regular nature hikes strengthen the heart [15,16,17,18].

Hiking is also linked to wellbeing. The wellbeing construct is not clear. Different conceptions of wellbeing reflect two types of happiness: hedonic and eudaimonic. Hedonic wellbeing can be defined as the presence of positive affect and the absence of negative affect. In turn, eudaimonic wellbeing is focused on an individual’s ability to realize their unique potential [19]. The eudaimonic approach suggests that wellbeing is more than just pleasure attainment and pain avoidance. Therefore, some researchers have proposed different complex models of wellbeing [20]. Seligman proposed the PERMA model of wellbeing. This model represents five core elements of wellbeing: positive emotions, engagement, relationships, meaning, and accomplishments [21]. In turn, Ryff [22] developed a model consisting of six core dimensions of psychological wellbeing: autonomy, environmental mastery, personal growth, positive relations with others, purpose in life, and self-acceptance. Diener et al. proposed the DRAMMA model of wellbeing, incorporating distraction, autonomy, challenges, relevance, and attachment [23].

Psychological wellbeing focuses on human resources or strengths. First-order positive psychological resources include hope, efficacy, resilience, and optimism. These four resources are included as a combined construct to form what Luthans and his coworkers referred to as positive psychological capital. Luthans claimed that “when these four resources are combined, they form, and have been empirically supported, as a higher-order core construct based on the shared commonalities of the four first-order constructs and their unique characteristics” p. 7 [24]. Research has shown that psychological capital is a key individual characteristic in the wellbeing of individuals [25].

Psychological wellbeing can be enhanced. Performing acts of kindness, expressing gratitude, and practicing forgiveness can improve wellbeing [26,27,28]. Psychological interventions such as cognitive behavioral (CB)-based approaches can also improve wellbeing [29,30]. Furthermore, interventions such as mindfulness training can be useful [31]. 

Research has indicated that hiking can also be effective in increasing wellbeing. For example, short walks surrounded by nature can lead to a significant increase in positive mood compared to walks in an urban environment [32,33,34]. Repeated hiking in a natural environment supports authenticity, autonomy, satisfaction with life, and a sense of meaning [35,36]. On the one hand, hiking promotes a reduction in stress, depression, internal anxiety, tension, anger, and even rumination [37,38,39]. On the other hand, hiking leads to the growth of positive emotions such as satisfaction or pleasure, as well as promotes resilience and the use of more effective coping strategies [40,41,42,43]. Hiking provides opportunities and places for social relations that lead to increased social connectedness and social cohesion [44,45].

Physically engaging in nature improves motor skills, efficacy, or self-esteem, as well as increases emotional intelligence and personal responsibility. This form of activity is also important for strong aesthetic and even transcendent experiences. The natural landscapes are those that cause admiration of the beauty and power of nature [46,47]. Contact with nature can also lead hikers to feel part of a larger project that goes beyond individual life [48]. Furthermore, hikers relaxing in close natural surroundings often return to the same places where they experienced something exciting. In this way, a specific identity of the place is formed that can increase the wellbeing of the hiker [49,50].

The above studies most often investigated the consequences of outdoor recreation for wellbeing. In other words, researchers aimed to determine how wellbeing changes when in contact with the natural environment. On the other hand, there is a lack of research on the everyday wellbeing of people undertaking recreation in the wilderness. Several studies analyzed the personality and temperament traits of recreationists, as well as self-efficacy or coping [51,52]. However, research on the mental wellbeing of recreationists is scarce.

Hence, the aim of the current research was to understand the daily wellbeing profiles of people engaging in mountain hiking. Another goal of the study was to distinguish types of mountain hikers depending on their wellbeing profiles and the type of adventures they undertake in the natural environment.

## 2. Method

### 2.1. Participants

A total of 242 participants (118 women and 124 men) from Poland took part in this research (M = 23.50; SD = 4.40). In total, 74.50% of the participants were from cities and 25.50% were from villages. All participants had at least a secondary level of education.

The respondents practiced hiking in the following Polish mountain ranges: The Tatras (62%), the Beskids (79%), and the Sudetes (63%). The hikers practiced hiking in the summer (100%), in the autumn (37%), in the winter (46%), and in the spring (58%).

The sums of percentages are higher than 100 because respondents practiced hiking in more than one mountain range and season.

### 2.2. Procedure

The study was approved by the University of Szczecin Institutional Review Board (14/2019; date of approval: 6 December 2019). I contacted mountain hikers through students, mountain tourism organizations, hiking associations, Nordic walking clubs, etc. The leaders from these organizations, associations, or clubs informed hikers about my research program. Mountain hikers who were interested in participating in the research clicked on a survey link. The hikers filled in questionnaires and sent them back to the author. Participation in the research was voluntary and anonymous.

Data collection took place between September and December 2021. The participants needed about 25 min to complete the questionnaires.

All of the participants were selected on the basis of the following criteria: (a) they had more than 1 year of experience in mountain hiking; (b) they were highly interested in participating in the research; (c) they were mountain hikers between 18 and 26 years old (I was looking for young hikers because they undertake harder adventures than other age groups [53]); (d) they practiced mountain hiking for at least 7 days per year.

Among the 258 interested outdoor recreationists, 16 did not meet the eligibility criteria. The exclusion criteria were (a) an age below 18 or above 26 years old, and (b) incidental mountain hiking (e.g., less than 1 week per year).

### 2.3. Materials

#### 2.3.1. Oxford Happiness Questionnaire (OHQ)

The Oxford Happiness Questionnaire (OHQ) [54] is a 29-item scale with all items included in one factor. The Polish version of the OHQ includes two subscales: general satisfaction with life (*Cronbach’s α* = 0.88) and control of life (*Cronbach’s α* = 0.82) Responses are rated on a six-point Likert scale ranging from 1 (strongly disagree) to 6 (strongly agree) [55].

#### 2.3.2. Meaning of Life Questionnaire (MLQ) 

The MLQ [56] is a nine-item scale with two subscales: presence of meaning in life (*Cronbach’s α* = 0.86) and search for meaning in life (*Cronbach’s α* = 0.72). The Polish adaptation was presented in [57]. Responses are rated on a seven-point Likert scale ranging from 1 (absolutely true) to 7 (absolutely untrue).

#### 2.3.3. Positive and Negative Affect Schedule (PANAS) 

The PANAS [58] is a 20-item scale with two subscales: positive affect (PA) and negative affect (NA). The correlation coefficients are 0.73 for positive affect and 0.90 for negative affect. The Polish adaptation was presented in [59]. Responses are rated on a five-point Likert scale ranging from 1 (very slightly or never) to 5 (very much).

#### 2.3.4. The Temporal Satisfaction with Life Scale

The Temporal Satisfaction with Life Scale [60] is a 15-item self-report instrument intended to diagnose the respondent’s past (*Cronbach’s α* = 0.81), present (*Cronbach’s α* = 0.79), and future life satisfaction (*Cronbach’s α* = 0.81). Responses are rated on a seven-point Likert scale ranging from 1 (absolutely untrue) to 7 (absolutely true). The Polish adaptation was presented in [61].

#### 2.3.5. Hope Scale

The Hope Scale [62] is a 12-item scale that measures the level of hope. The Hope Scale consists of two subscales: agency (e.g., measures one’s goal-directed energy to pursue one’s goals) (*Cronbach’s α* = 0.82) and pathway (e.g., measures one’s extent of creating ways to achieve one’s goal) (*Cronbach’s α* = 0.72). In this study, the agency subscale was used. Responses are rated on a seven-point Likert scale ranging from 1 (definitely false) to 7 (definitely true). The Polish adaptation was presented in [63].

#### 2.3.6. General Self-Efficacy Scale

The General Self-Efficacy Scale [64] consists of 10 statements, with all items included in one factor. It diagnoses an individual’s general self-efficacy beliefs in the face of difficult situations (*Cronbach’s α =* 0.85). Responses are rated on a four-point Likert scale ranging from 1 (not at all true) to 4 (exactly true). The Polish adaptation was presented in [65].

#### 2.3.7. Ego Resiliency Scale

The Ego Resiliency Scale [66] consists of 14 items. It measures resiliency in different situations (*Cronbach’s α* = 0.78). Responses are rated on a four-point Likert scale ranging from 1 (does not apply at all) to 4 (applies very strongly). The Polish adaptation was presented in [67].

#### 2.3.8. Life Orientation Test Revised (LOT-R)

The Life Orientation Test Revised (LOT-R) [68] is a 10-item unidimensional scale (*Cronbach’s α =* 0.73). The Polish adaptation was presented in [69]. The questionnaire measures dispositional optimism. Responses are rated on a five-point Likert scale ranging from 0 (strongly disagree) to 4 (strongly agree).

#### 2.3.9. Adventure -Seeking Behavior Scale 

The Adventure-Seeking Behavior Scale [70] was developed to assess an individual’s stimulating behavior in a natural environment. The ABSS is an eight-item scale that determines an individual’s willingness to engage in a variety of outdoor recreations despite water, gravity, and weather risks (*Cronbach’s α =* 0.80).

### 2.4. Statistical Analysis

All tests were performed using Statistica 13.0 (StatSoft, Krakow, Poland). Different types of cluster analysis were used to extract clusters (profiles of mountain hikers): k-means clustering method and expectation (E)–maximization (M) clustering method.

The k-means clustering method was used in this study to extract the basic clusters of wellbeing of participants who undertook different forms of adventure in close contact with the natural environment. The main goal of the k-means clustering method is to extract clusters such that observations belonging to the same cluster are very similar or homogeneous and observations belonging to different clusters are different or heterogeneous [71].

EM cluster analysis was used to segment participants in this study. The general purpose of EM clustering is to detect clusters in examples and to assign participants to these clusters. The advantage of the EM method is that (unlike the classic method of cluster analysis) it enables the handling of not only quantitative variables (seeking adventure), but also qualitative ones (profiles of psychological functioning). This method also allows for the automatic identification of the optimal number of groups [71].

## 3. Results

### 3.1. Profiles of The Oxford Happiness Questionnaire in the Group of Mountain Hikers

The first step when using the k-means cluster method is to indicate the number of clusters that will be generated in the final solution. Therefore, I checked whether the variables general satisfaction with life and control of life could be grouped into two clusters. Results of the k-means cluster method indicated that this solution was unsatisfactory (higher variance between groups than variance within any single group is an important criterion in extracting clusters [71]). In the case of general satisfaction with life, this criterion was not met for two clusters. The k-means cluster method showed that the three-cluster model met this criterion. In this model, variance between the groups is higher than variance within the groups for both variables: general satisfaction with life and control of life. This means that participants in particular clusters obtained similar results for these two variables, and they were different from the results of subjects from other clusters (see Table 1).

**Figure 1:**
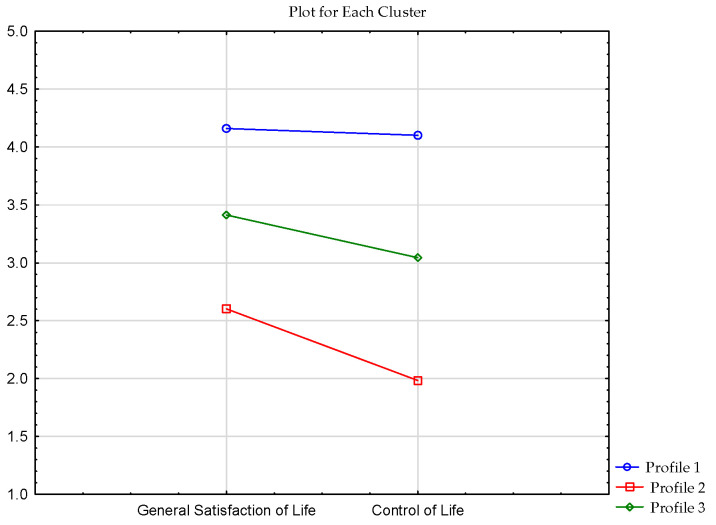
Clusters of OHQ in the sample of respondents.

Cluster analysis revealed three clusters. The first cluster contained 98 individuals who had high scores on both scales (i.e., a high score on the general satisfaction with life subscale and a high score on control of life subscale). The second cluster comprised 32 respondents who scored low on the general satisfaction with life subscale and low on the control of life subscale. The last cluster was composed of 112 respondents who received averages scores on both scales (see Figure 1).

### 3.2. Profiles of Meaning of Life in the Group of Mountain Hikers

**Figure 2:**
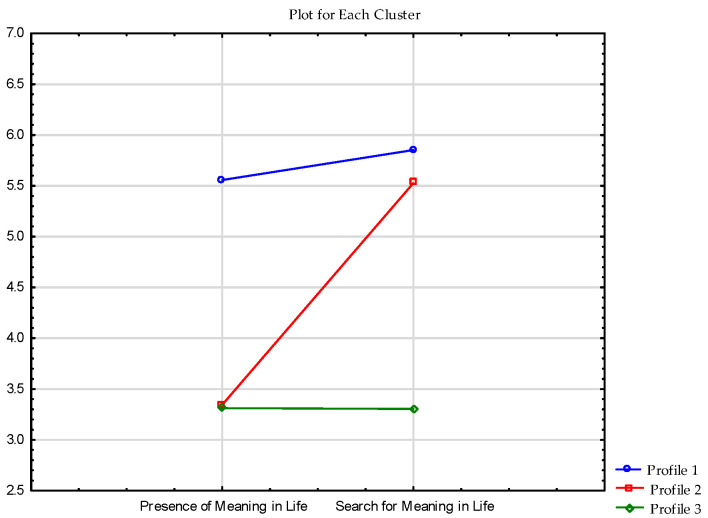
Clusters of MLQ scale in the sample of respondents.

In next step, I analyzed profiles of the meaning of life in the group of outdoor recreationists. The results are presented in Table 2 and Figure 2.

Cluster analysis revealed three clusters. The first cluster contained 114 individuals who had high scores on both scales (i.e., a high score on the presence of meaning in life subscale and a high score on search for meaning in life scale). The second cluster comprised 91 respondents who scored average on the Presence of meaning in life and scored high on the search for meaning scale. The last cluster was composed of 37 respondents who received averages scores on both scales (see Figure 2).

### 3.3. Positive and Negative Affect in the Group of Mountain Hikers

**Figure 3:**
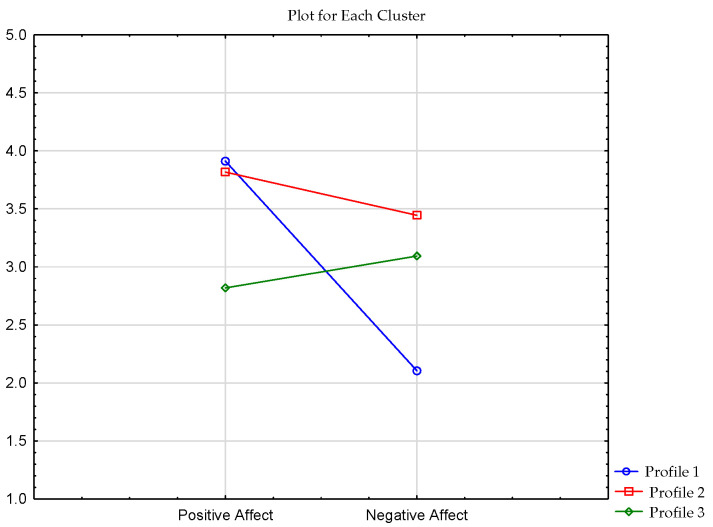
Clusters of PANAS Scale in the sample of respondents.

Another step included the analysis of profiles for PANAS scale (positive and negative affect). The results are presented in Table 3 and Figure 3.

The first cluster comprised 94 respondents who scored high for positive affect and low for negative affect. The second cluster contained 72 recreationists who had high scores on both PANAS scales. The last cluster was composed of 76 respondents who received a lower score for positive affect and a higher score for negative affect.

### 3.4. Temporal Satisfaction with Life in the Group of Mountain Hikers

The results of cluster analysis for the Temporal Satisfaction with Life Scale (see Table 4).

**Figure 4:**
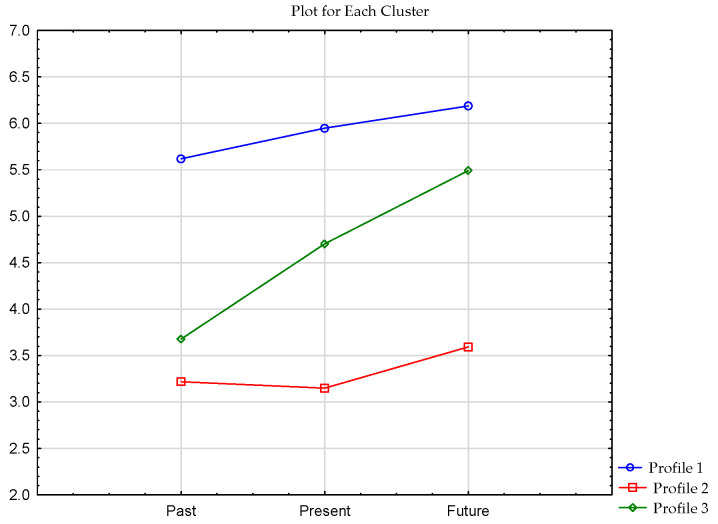
Clusters of TSLQ scale in the sample of respondents.

The first cluster comprised 85 respondents who scored high on all three scales of temporal satisfaction in life. The second cluster contained 58 individuals who had average scores for temporal satisfaction with life. The last cluster was composed of 99 respondents who received average scores for the past and present satisfaction scales but a high score for the future satisfaction scale (see Figure 4).

### 3.5. Psychological Capital in the Group of Mountain Hikers

The final step included an analysis of psychological capital in the group of outdoor recreationists. Psychological capital consisted of the following variables: hope, self-efficacy, resilience, and optimism (see Table 5).

**Figure 5:**
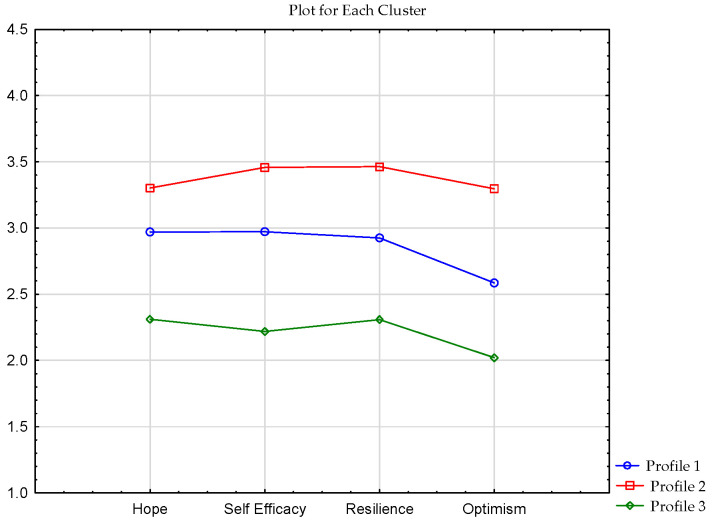
Clusters of psychological capital in the sample of respondents.

Cluster analysis revealed three clusters. The first cluster contained 136 individuals who had averages scores on all Psychological Capital scales. The second cluster comprised 79 respondents who scored high on the Psychological Capital scales. The last cluster consisted of 27 respondents who received low scores on all Psychological Capital scales (see Figure 5).

### 3.6. Typology of Mountain Hikers Based on Their Wellbeing Profiles and Adventure-Seeking Behavior Scale Scores

The results of the research on the psychological profiles of mountain hikers were the basis for the further statistical analyses presented below. The goal was to understand the psychological wellbeing profiles of people with different scores on the Adventure-Seeking Behavior Scale. 

EM (expectation–maximization) cluster analysis was used to segment the participants in this study. The general purpose of EM clustering is to detect clusters in examples and assign those participants to the clusters. The advantage of the EM method is that (unlike the classic method of cluster analysis) it enables the handling of not only quantitative variables (seeking adventure), but also qualitative ones (profiles of psychological functioning). This method also allows for the automatic identification of the optimal number of groups [71].

EM cluster analysis revealed two types of mountain hikers with different scores on the *Adventure-Seeking Behavior Scale* (so-called *hard adventurers* (*n* = 100) and *soft adventurers* (*n* = 142)). The two groups of adventurers were also characterized by different profiles of psychological wellbeing (see Table 6).

The two types of mountain hikers differed in terms of subjective wellbeing profiles. Hard adventurers most often revealed the following wellbeing profiles: high level of life satisfaction and high level of life control, high level of meaning in life and high level of searching for new meaning in life, high level of positive emotions and low level of negative emotions, high level of satisfaction in various time perspectives (past, present, and future), and a high level of psychological capital. On the other hand, soft adventurers most often revealed an average level of satisfaction with life and average level of life control, average level of the meaning of life and high level of searching for the meaning of life, the average level of positive emotions and higher level of negative emotions, high level of satisfaction in a future perspective and average levels of satisfaction in past and present time perspectives, and average level of psychological capital.

## 4. Discussion

The conducted research was primarily aimed at distinguishing the wellbeing profiles of people undertaking mountain hiking. In turn, the separated wellbeing profiles and the Adventure-Seeking Behavior Scale score were the basis for the classification of types of mountain hikers.

The results of cluster analysis using the EM method indicated two main types of mountain hikers: hard adventure hikers and soft adventure hikers. Hard adventure hikers like being active in the natural environment despite various threats to their health or even life. The sources of these threats are water conditions, gravity conditions, or unfavorable weather conditions.

The research results indicated that hard adventure hikers were characterized by high mental wellbeing. They considered themselves in control of their own lives. In everyday life, they revealed a high level of positive emotions and a low level of negative emotions. Hard adventure hikers were satisfied with the past, present and future. They found meaning in their own lives, but also looked for new meanings. They had a high level of personal resources. This is in line with research outcomes that identified low levels of negative emotions and a high level of psychological potential in a group of risk takers in the wilderness [72,73,74].

The profile of hard adventure hikers included high levels of personal resources. Personal resources allow people to achieve personal aspirations, reducing different costs associated with the demanding conditions of environments. Personal resources promote subjective wellbeing [73] High levels of personal resources in the group of hard adventure hikers granted them more resources at their disposal to cope with dangers in the wilderness and allowed them to influence the outcomes of events, both positive and negative. To them, adventure in the wilderness could stimulate personal growth. This is in line with research outcomes that identified high levels of psychological capital in a group of adventurers [73].

In contrast the previous group, soft adventure hikers were more likely to have lower wellbeing. They also had lower confidence in control of their own life. Compared to hard adventure hikers, they also experienced negative emotions more often. The source of their greatest satisfaction was the future. They were less satisfied with the past and present. Less often, compared to hard adventure hikers, they had a sense of the presence of meaning in life; however, they also looked for new meanings. They had lower psychological capital scores compared to hard adventure hikers.

What is the significance of the obtained research results for functioning in the natural environment? What are consequences of these results for taking up challenges in the natural environment? These results suggest that hard adventure hikers may control stress more effectively than soft adventure hikers, and they may react significantly less often with negative emotions in the wilderness. This means that their personal safety may be less at risk in the wilderness than soft adventure hikers. Moreover, they may tend not to focus on the potential negative consequences of remaining in a threatening natural environment. Hard adventure hikers are optimistic persons with high levels satisfaction with life. However, one must remember that this may often be unrealistic optimism, which may lead to underestimation of the risk of death in the wilderness [75].

The results can also be interpreted using different complex models of wellbeing. The PERMA model emphasizes the importance of achievements in creating wellbeing [21]. Similarly, the DRAMMA model includes the component of challenges [23]. In turn, Ryff’s six-factor model of wellbeing describes environmental mastery as an important component of personal happiness [22]. Hard adventure recreation enables the achievement of challenging goals and the improvement of one’s own competences in a natural way; thus, its relationship with mental health and positive functioning seems obvious.

### 4.1. Limitations of the Study and Future Directions

An important limitation of the present study is that the participants in this study were young people. They were a select group and not a representative sample of the population. This fact limits the generalizability of the results. In future research, it would be important to assess not only young adults but also other groups of people.

Another important limitation of the present study is that information regarding the socioeconomic factors of participants was very limited. The findings likely apply to participants of different socioeconomic backgrounds, in terms of including educational attainment, profession, race and ethnicity, and household income. Future research should explain the role of socioeconomic status in the typology of outdoor recreationists.

The data were collected in Poland. This limits the generalizability of the results for people from other geographical regions. Therefore, in future research, it would be interesting to analyze the profiles of wellbeing in hikers from other countries.

In this study, only some of the variables of wellbeing were subjected to analysis. Future research might encompass other variables of wellbeing such as flow, flourishing, vitality, or the dimensions of wellbeing proposed by Ryff [22].

In the current study, the participants of the study were not diagnosed during outdoor recreation. In future studies, adventure participants could fill out the questionnaires during outdoor recreation. Furthermore, future research can examine participants before embarking on an adventure in the wilderness and after completing their adventure.

The current research focused only on one type of outdoor recreation (mountain hiking). This means that the results can only be applied to a very narrow population. Therefore, in future research, it would be interesting to analyze profiles of wellbeing in other recreationists such as skydivers, paragliders, or skiers.

An important limitation of the current study is that some differences between hard and soft adventurers were not very significant and should more be considered a trend.

### 4.2. Applications of Current Study

The research results showed that people who are looking for adventures in the natural environment are more often characterized by high wellbeing profiles. This knowledge can be an important argument for people who hesitate with regard to taking up challenges in the natural environment. Moreover, the obtained research results can be used in outdoor education. Information that an adventurer in the natural environment may be associated with higher wellbeing can be a valuable hint for people conducting adventure therapy or people who manage survival schools.

## 5. Conclusions

Researchers very often analyze the motivational or personality factors of outdoor recreationists. This perspective explains why adventurers explore the natural environment; however, it says little about the daily subjective wellbeing of these people. The present research indicated that two types of wellbeing profiles exist among outdoor recreationists: hard adventurers and soft adventurers. Distinguishing these types of recreationists can allow us to better understand the phenomenon of outdoor recreation.

## Figures and Tables

**Table 1:** Variance within and between groups for the *Oxford Happiness Questionnaire*: results of k-means cluster analysis.

Model	Variable	Variance between Group	df	Variance within Group	df	F	*p*
Two clusters	General satisfaction with life	53.14	1	58.09	240	219.55	0.01
	Control of life	97.64	1	64.26	240	364.63	0.01
Three clusters	General satisfaction with life	66.43	2	44.80	239	177.21	0.01
	Control of life	125.45	2	36.46	239	411.17	0.01

**Table 2:** Variance within and between groups for the *Meaning of Life Questionnaire (MLQ)*: results of k-means cluster analysis.

Model	Variable	Variance between Group	df	Variance within Group	df	F	*p*
Two clusters	Presence of meaning in life	320.57	1	182.55	240	421.44	0.01
	Search for meaning in life	49.85	1	282.27	240	42.38	0.01
Three clusters	Presence of meaning in life	297.34	2	205.81	239	172.62	0.01
	Search for meaning in life	186.57	2	145.54	239	153.18	0.01

**Table 3:** Variance within and between groups for PANAS scale: results of k-means cluster analysis.

Model	Variable	Variance between Group	df	Variance within Group	df	F	*p*
Two clusters	Positive affect	31.20	1	74.36	240	100.70	0.01
	Negative affect	73.23	1	71.46	240	245.96	0.01
Three clusters	Positive affect	58.05	2	47.51	239	146.01	0.01
	Negative affect	81.88	2	62.81	239	155.78	0.01

**Table 4:** Variance within and between groups for *the Temporal Satisfaction with Life Scale*: results of k-means clustering analysis.

Model	Variable	Variance between Group	df	Variance within Group	df	F	*p*
Two clusters	Past satisfaction	162.15	1	301.35	240	129.13	0.01
	Present satisfaction	256.04	1	173.66	240	353.85	0.01
	Future satisfaction	169.93	1	218.73	240	186.44	0.01
Three clusters	Past satisfaction	253.70	2	209.81	239	144.49	0.01
	Present satisfaction	270.91	2	158.79	239	192.52	0.01
	Future satisfaction	239.81	2	148.85	239	192.52	0.01

**Table 5:** Variance within and between groups for Psychological Capital Scale: results of k-means clustering analysis.

Model	Variable	Variance between Group	df	Variance within Group	df	F	*p*
Two clusters	Hope	13.78	1	25.83	240	1428.08	0.01
	Self-efficacy	26.16	1	36.67	240	171.20	0.01
	Resilience	24.21	1	34.23	240	169.75	0.01
	Optimism	26.96	1	49.75	240	130.04	0.01
Three clusters	Hope	20.15	2	19.46	239	123.71	0.01
	Self-efficacy	32.64	2	30.20	239	129.14	0.01
	Resilience	30.35	2	28.09	239	129.12	0.01
	Optimism	41.58	2	35.13	239	141.45	0.01

**Table 6:** Profiles of wellbeing in the group of soft and hard adventurers.

Profile	Soft Adventurers	Hard Adventurers	Number
The Oxford Happiness Questionnaire			
Profile 1: high satisfaction with life/high control of life	8	90	98
Profile 2: lower satisfaction with life/lower control of life	32	0	32
Profile 3: average satisfaction with life/average control of life	102	10	112
Chi^2^ = 174.13, *p* < 0.01; G^2^ = 103.05, *p* < 0.01			
Meaning of Life Questionnaire			
Profile 1: high presence of meaning/high search for meaning	30	84	114
Profile 2: average presence of meaning/high search for meaning	77	14	91
Profile 3: average presence of meaning/average search for meaning	35	2	37
Chi^2^ = 94.17, *p* < 0.01; G^2^ = 205.34, *p* < 0.01			
Positive and Negative Affect Scale			
Profile 1: high positive affect/low negative affect	16	78	94
Profile 2: high positive affect/ high negative affect	50	22	72
Profile 3 average positive affect/ high negative affect	76	0	76
Chi^2^ = 124.23, *p* < 0.01; G^2^ = 153.75, *p* < 0.01			
Temporal Satisfaction with Life			
Profile 1: high in past/high in present/high in future	19	66	85
Profile 2: average in past/average in presence/average in future	56	2	58
Profile 3: average in past/average in present/high in future	67	32	99
Chi^2^ = 83.87, *p* < 0.01; G^2^ = 95.83, *p* < 0.01			
Psychological Capital			
Profile 1: average hope/average self-efficacy/average resilience/average optimism	97	39	136
Profile 2: high hope/high self-efficacy/high resilience/ high optimism	18	61	79
Profile 3: lower hope/lower self-efficacy/lower resilience/ lower optimism	27	0	27
Chi^2^ = 69.95, *p* < 0.01; G^2^ = 80.37, *p* < 0.01			

## Data Availability

The datasets used during this study are available from the author.

## References

[1] Chhetri P., Arrowsmith C., Jackson M. (2004). Determining hiking experiences in nature-based tourist destinations. Tour. Manag..

[2] Rodrigues A., Kastenholz E., Rodrigues A. (2010). Hiking as a wellness activity. An exploratory study of hiking tourists in Portugal. J. Vacat. Mark..

[3] Bichler B.F., Peters M. (2021). Soft adventure motivation: An exploratory study of hiking tourism. Tour. Rev..

[4] Ewert A., Gilbertson K., Luo Y.C., Voight A. (2013). Beyond because it’s there. Motivations for pursuing adventure recreational activities. J. Leis. Res..

[5] Nordbø I., Prebensen N.K. (2015). Hiking as mental and physical experience. Adv. Hosp. Leis..

[6] Kortenkamp K.V., Moore C.F., Sheridan D.P., Ahrens E.S. (2017). No hiking beyond this point! Hiking risk prevention recommendations in peer-reviewed literature. J. Outdoor Recreat. Tour..

[7] Próchniak P., Próchniak A. (2021). Future-Oriented Coping with Weather Stress among Mountain Hikers: Temperamental Personality Predictors and Profiles. Behav. Sci..

[8] Schöffl V., Morrison A.B., Schwarz U., Schöffl I., Küpper T. (2010). Evaluation of injury and fatality risk in rock and ice climbing. Sports Med..

[9] She S., Tian Y., Lu L., Eimontaite I., Xie T., Sun Y. (2019). An Exploration of Hiking Risk Perception: Dimensions and Antecedent Factors. Int. J. Environ. Res. Public Health.

[10] Hill B.J. (1995). A Guide to Adventure Travel. Parks Recreat..

[11] Millington K., Locke T., Locke A. (2001). Adventure travel. Travel Tour. Anal..

[12] Buckley R. (2018). To Analyze Thrill, Define Extreme Sports. Front. Psychol..

[13] Molokáč M., Hlaváčová J., Tometzová D., Liptáková E. (2022). The Preference Analysis for Hikers’ Choice of Hiking Trail. Sustainability.

[14] Próchniak P. (2020). Coping with Stress and Pain in Hard and Soft Adventure Mountain Athletes. Ann. Psychol..

[15] Eigenschenk B., Thomann A., McClure M., Davies L., Gregory M., Dettweiler U., Inglés E. (2019). Benefits of Outdoor Sports for Society. A Systematic Literature Review and Reflections on Evidence. Int. J. Environ. Res. Public Health.

[16] Murphy M.H., Nevill A.M., Murtagh E.M., Holder R.L. (2007). The effect of walking on fitness, fatness and resting blood pressure: A meta-analysis of randomised, controlled trials. Prev. Med..

[17] Hansmann R., Hug S.-M., Seelanda K. (2007). Restoration and stress relief through physical activities in forests and parks. Urban For. Urban Green..

[18] Hanson S., Jones A. (2015). Is there benefit that walking groups have health benefits? A systematic review and meta-analysis. Br. J. Sports Med..

[19] Diener E., Suh E.M., Lucas R.E., Smith H.L. (1999). Subjective well-being: Three decades of progress. Psychol. Bull..

[20] Denier E., Ryan K. (2009). Subjective wellbeing: A general overview. S. Afr. J. Psychol..

[21] Seligman M.E. (2012). Flourish: A Visionary New Understanding of Happiness and Well-Being.

[22] Ryff C.D. (2014). Psychological well-being revisited: Advances in the science and practice of eudaimonia. Psychother. Psychosom..

[23] Newman D.B., Tay L., Diener E. (2014). Leisure and subjective well-being: A model of psychological mechanisms as mediating factors. J. Happiness Stud. Interdiscip. Forum Subj. Well-Being.

[24] Luthans F., Youssef-Morgan C.M. (2017). Psychological Capital: An Evidence-Based Positive Approach. Annu. Rev. Organ. Psychol. Organ. Behav..

[25] Luthans F., Youssef C.M. (2007). Emerging Positive Organizational Behavior. J. Manag..

[26] Huppert F.A. (2009). Psychological well-being: Evidence regarding its causes and consequences. Appl. Psychol. Health Well-Being.

[27] Kubzansky L.D., Huffman J., Boehm J., Hernandez R. (2018). Positive psychological well-being and cardiovascular disease: JACC health promotion series. J. Am. Coll. Cardiol..

[28] Mathers N. (2016). Compassion and the science of kindness: Harvard Davis Lecture 2015. Br. J. Gen. Pract..

[29] Unsworth K.L., Mason C.M. (2012). Help yourself: The mechanisms through which a self-leadership intervention influences strain. J. Occup. Health Psychol..

[30] Bolier L., Ketelaar S.M., Nieuwenhuijsen K., Smeets O., Gärtner F.R., Sluiter J.K. (2014). Workplace mental health promotion online to enhance well-being of nurses and allied health professionals: A cluster-randomized controlled trial. Internet Interv..

[31] Sakuraya A., Imamura K., Watanabe K., Asai Y., Ando E., Eguchi H., Nishida N., Kobayashi Y., Arima H., Iwanaga M. (2020). What Kind of Intervention Is Effective for Improving Subjective Well-Being Among Workers? A Systematic Review and Meta-Analysis of Randomized Controlled Trials. Front. Psychol..

[32] Mayer F.S., McPherson Frantz C. (2004). The Connectedness to nature scale: A measure of individuals’ feeling in community with nature. J. Environ. Psychol..

[33] McMahan E.A., Estes D. (2015). The effect of contact with natural environments on positive and negative affect: A meta-analysis. J. Posit. Psychol..

[34] Nisbet E.K., Zelenski J.M. (2011). Underestimating nearby nature: Affective forecasting errors obscure the happy path to sustainability. Psychol. Sci..

[35] Korpela K., Borodulin K., Neuvonen M., Paronen O., Tyrväinen L. (2014). Analyzing the mediators between nature-based outdoor recreation and emotional well-being. J. Environ. Psychol..

[36] Cervinka R., Roderer K., Hefler E. (2012). Are nature lovers happy? On various indicators of well-being and connectedness with nature. J. Health Psychol..

[37] Bodin M., Hartig T. (2003). Does the outdoor environment matter for psychological restoration gained through running?. Psychol. Sport Exerc..

[38] Crust L., Henderson H., Middleton G. (2013). The acute effects of urban green and countryside walking on psychological health: A field-based study of green exercise. Int. J. Sport Psychol..

[39] Bratman G.N., Hamilton J.P., Hahn K.S., Daily G.C., Gross J.J. (2015). Nature experience reduces rumination and subgenual prefrontal cortex activation. Proc. Natl. Acad. Sci. USA.

[40] Clough P., Houge McKenzie S., Mallabon M.E., Brymer E. (2016). Adventurous physical activity environments: A mainstream intervention for mental health. Sports Med..

[41] Levin B.J., Taylor J. (2011). Depression, Anxiety, and Coping in Surfers. J. Clin. Sport Psychol..

[42] MacGregor A.L., Woodman T., Hardy L. (2014). Risk is good for you: An investigation of the processes and outcomes associated with high-risk sport. J. Exerc. Mov. Sport.

[43] Pierskalla C.D., Lee M.A., Stein T.V., Anderson D.H., Nickerson R. (2004). Understanding relationships among recreation opportunities: A meta analysis of nine studies. Leis. Sci..

[44] Breunig M., O’Connell T.S., Todd S., Anderson L., Young A. (2010). The Impact of Outdoor Pursuits on College Students’ Perceived Sense of Community. J. Leis. Res..

[45] Dorsch T.E., Maxey M., Richards A.R. (2016). The effect of an outdoor recreation program on individuals with disabilities and their family members: A case study. Ther. Recreat. J..

[46] Frumkin H., Bratman G.N., Breslow S.J., Cochran B., Kahn P.H., Lawler J.J., Wood S.A. (2017). Nature contact and human health: A research agenda. Environ. Health Perspect..

[47] Keltner D., Haidt J. (2003). Approaching awe, a moral, spiritual, and aesthetic emotion. Cogn. Emot..

[48] Shiota M.N., Keltner D., Mossman A. (2007). The nature of awe: Elicitors, appraisals, and effects on self-concept. Cogn. Emot..

[49] Houge Mackenzie S., Hodge K., Boyes M. (2013). The multiphasic and dynamic nature of flow in adventure experiences. J. Leis. Res..

[50] Twigger-Ross C.L., Uzzell D.L. (1996). Place and identity processes. J. Environ. Psychol..

[51] Llewellyn D.J., Sanchez X., Asghar A., Jones G. (2008). Self-efficacy, risk taking and performance in rock climbing. Personal. Individ. Differ..

[52] Brymer E., Feletti F., Monasterio E., Schweitzer R. (2020). Editorial: Understanding Extreme Sports: A Psychological Perspective. Frontiers Psychology..

[53] Steinberg L. (2010). A dual systems model of adolescent risk-taking. Dev. Psychobiol..

[54] Argyle M., Hills P. (2002). The Oxford Happiness Questionnaire: A compact scale for the measurement of psychological well-being. Personal. Individ. Differ..

[55] Kołodziej-Zaleska A., Przybyła-Basista H. (2018). Dobrostan psychiczny i jego pomiar za pomocą polskiej wersji Oksfordzkiego Kwestionariusza Szczęścia. [Psychological well-being and its measurement with a polish version of the Oxford Happiness Questionnaire]. Czas. Psychol.-Psychol. J..

[56] Steger M.F., Frazier P., Oishi S., Kaler M. (2006). The Meaning in Life Questionnaire: The presence of and search for meaning in life. J. Couns. Psychol..

[57] Kossakowska M., Kwiatek P., Stefaniak T. (2013). Sens w życiu. Polska wersja kwestionariusza MLQ (Meaning of Life Questionnaire). [Meaning in Life. Polish Version of MLQ]. Psychol. Jakości Życia.

[58] Watson D., Clark L.A., Tellegen A. (1988). Development and validation of brief measures of positive and negative affect: The PANAS scales. J. Personal. Soc. Psychol..

[59] Brzozowski P. (2010). Skala Uczuć Pozytywnych i Negatywnych SUPIN. Polska Adaptacja Skali PANAS Davida Watsona i Lee Anny Clark. Podręcznik [The Scale of Positive and Negative Feelings SUPIN. Polish Adaptation of the PANAS Scales of David Watson and Lee Anna Clark. Handbook].

[60] Pavot W., Diener E., Suh E. (1998). The Temporal Satisfaction with Life Scale. J. Personal. Assess..

[61] Próchniak P. (2022). Temporal Satisfaction with Life Scale. Polish adaptation. Ann. Psychol..

[62] Snyder C.R. (1995). Conceptualizing, measuring, and nurturing hope. J. Couns. Dev..

[63] Łaguna M., Trzebiński J., Zięba M. (2005). Kwestionariusz Nadziei na Sukces. Podręcznik. [The Hope for Success Questionnaire. Handbook].

[64] Schwarzer R. (1998). General Perceived Self-Efficacy in 14 Cultures, An Electronic Volume. Produced for the European Health Psychology Society.

[65] Juczyński Z. (2000). Poczucie własnej skuteczności–teoria i pomiar. [Self-Efficacy-theory and measuring]. Acta Univ. Lodz..

[66] Block J., Kremen A.M. (1996). IQ and ego-resiliency: Conceptual and empirical connections and separateness. J. Personal. Soc. Psychol..

[67] Kaczmarek Ł. (2011). Skala Sprężystości Psychicznej–polska adaptacja Ego Resiliency Scale. [Adaptation and Validation of Ego Resiliency Scale into Polish]. Czas. Psychol.-Psychol. J..

[68] Carver C.S., Scheier M.F., Segerstrom S.C. (2010). Optimism. Clin. Psychol. Rev..

[69] Juczyński Z. (2001). Narzędzia Pomiaru w Promocji i Psychologii Zdrowia [Measures in Promotion and Health Psychology].

[70] Próchniak P. (2017). Adventure Behavior Seeking Scale. Behav. Sci..

[71] Jung Y.G., Kang M.S., Heo J. (2014). Clustering performance comparison using K-means and expectation maximization algorithms. Biotechnol. Biotechnol. Equip..

[72] Egan S., Stelmack M.R. (2003). A personality profile of Mount Everest climbers. Personal. Individ. Differ..

[73] Próchniak P., Próchniak A. (2022). Personal Resources of Winter and Summer Hikers Visiting the Tatra National Park, Poland. Int. Environ. Res. Public Health.

[74] Tok S. (2011). The big five personality traits and risky sport participation. Soc. Behav. Personal..

[75] Weinstein N.D. (1980). Unrealistic optimism about future life events. J. Personal. Soc. Psychol..

